# Advancing Healthcare Equity in Nephrology: Addressing Racial and Ethnic Disparities in Research Trials and Treatment Strategies

**DOI:** 10.7759/cureus.56913

**Published:** 2024-03-25

**Authors:** Yaqub Nadeem Mohammed, Sakshi Khurana, Amit Gulati, Zubair Rahaman, Abhi C Lohana, Ramchandani Santosh

**Affiliations:** 1 Internal Medicine, Homer Stryker M.D. School of Medicine, Western Michigan University, Kalamazoo, USA; 2 Radiology, New York Presbyterian/Columbia University Irving Medical Center, New York, USA; 3 Cardiology, Icahn School of Medicine at Mount Sinai, New York, USA; 4 Internal Medicine, Jacobs School of Medicine and Biomedical Sciences, University at Buffalo, Buffalo, USA; 5 Internal Medicine, Camden Clark Medical Center, West Virginia University, Parkersburg, USA; 6 Internal Medicine/Nephrology, Lehigh Valley Hospital, Allentown, USA

**Keywords:** advancement in nephrology outcomes, nephrology, healthcare equity, chronic kidney disease, gender and racial disparity in nephrology

## Abstract

Within the healthcare sector, especially in the field of nephrology, the matter of gender and racial inequalities continues to be a critical concern that requires immediate focus. Women, particularly those of underrepresented racial groups, face significant challenges due to a lack of representation in research studies, leading to a deficit in knowledge about how kidney diseases affect them differently. These challenges are exacerbated by systemic biases in the healthcare system, which manifest in both gender and racial dimensions, hindering access to and the quality of care for kidney diseases. Addressing these complex disparities requires a recalibration of risk stratification models to include both gender- and race-specific factors and a transformation of healthcare policies to facilitate a more inclusive and sensitive approach. Essential to this transformation is the empowerment of women of all races to actively participate in their healthcare decisions and the strengthening of support systems to help them navigate the complexities of the healthcare environment. Furthermore, education programs must be designed to be culturally competent and address the unique needs and concerns of women across different racial backgrounds. Promoting a collaborative patient-provider relationship is crucial in fostering an environment where equity, dignity, and respect are at the forefront. The path to equitable nephrology care lies in a concerted, collective action from researchers, healthcare providers, policymakers, and patients, ensuring that every individual receives the highest standard of care, irrespective of gender or race.

## Editorial

In the healthcare sector, and particularly within the specialty of nephrology, the issue of gender and racial disparities remains a pressing issue that demands urgent attention. Nephrology, which focuses on the study, diagnosis, and treatment of kidney diseases, is an area where significant gender- and racial-based disparities are evident, not only within the scope of research but also in the delivery of patient care and the health outcomes achieved [1]. Addressing these disparities is imperative, not merely as a question of equity but as a vital component in the advancement of medical science and the assurance of optimal health outcomes for every individual.

One of the critical factors contributing to gender disparities in nephrology is the stark underrepresentation of women in research studies [1,2]. Although women constitute nearly half of the world's population, they are conspicuously underrepresented in clinical trials and research initiatives related to kidney diseases [2]. The underrepresentation of women, especially those from racially marginalized groups, in nephrology research critically undermines the understanding of disease progression and treatment efficacy across diverse populations [3]. Gender-specific factors such as hormonal fluctuations, pregnancy, and menopause, coupled with the intersectional issues of race, significantly influence kidney health and disease outcomes [4]. These crucial elements are too often overlooked in research settings, resulting in healthcare that may not be fully attuned to the physiological and sociocultural variations among women. The consequent lack of targeted investigation into these intersections deepens the disparities in healthcare outcomes for women with kidney disorders. To bridge both gender and racial gaps in research, a deliberate and inclusive approach is recommended. This approach must aim to expand our understanding of kidney diseases through a lens that acknowledges the full spectrum of gender and racial diversities. Developing treatment strategies that cater to this diversity is fundamental for achieving equitable healthcare. Therefore, reinforcing the inclusion of women from all racial backgrounds in nephrology research is a critical step toward realizing comprehensive equality and improved health outcomes in healthcare.

The inclusion of women in nephrology research is a critical step but does not single-handedly resolve the deep-seated issues of gender and racial biases in medical research. Historically, clinical research has disproportionately centered on male participants, which has led to significant gaps in the collective understanding of different manifestations of diseases as they affect women, particularly women of color [5,6]. This discrepancy hampers the development of diagnostic tools, treatment plans, and clinical guidelines that accurately reflect the health needs of all women. To address these shortcomings, it is imperative to shift toward a research paradigm that not only seeks the participation of a diverse group of women but also conducts a thorough analysis of the nuances introduced by gender and race. Embedding gender- and race-specific factors into research methodologies is essential for the creation of treatment protocols that resonate with the diverse experiences of women. This progressive approach is pivotal for cultivating nuanced and individualized management of kidney diseases across all demographics, ensuring healthcare practices are equitable. Bridging these knowledge gaps is a critical scientific endeavor and a vital move toward a future where high-quality healthcare is universally accessible and sensitive to the full spectrum of gender and racial diversities.

Gender and racial disparities in CKD management and outcome

The management and outcomes of chronic kidney disease (CKD) are significantly influenced by gender and racial disparities, which disproportionately impact women, especially those from underrepresented racial and ethnic groups [4]. Studies have highlighted women, and more acutely women of color, are less likely to receive referrals for specialized care or undergo kidney transplantation compared to their male counterparts [5]. This inequality is rooted in a complex web of provider bias, socioeconomic disparities, gender-specific variations in disease manifestation, and racial prejudice [7]. These factors collectively contribute to a healthcare system that fails to serve all its patients equally.

Implicit biases among healthcare providers, which may be influenced by both gender and racial stereotypes, play a significant role in perpetuating these disparities. These biases can skew clinical judgments, leading to differentiated treatment recommendations that disadvantage women, particularly those belonging to racial or ethnic minorities [7,8]. The situation is further exacerbated by socioeconomic factors that disproportionately affect women of color, such as financial instability, limited access to healthcare resources, and gaps in health insurance coverage. These barriers are formidable, hindering the ability of these women to receive timely and adequate care for kidney diseases [8]. Therefore, addressing the intertwined issues of gender and racial disparities is essential for achieving equitable healthcare and improving outcomes for all individuals suffering from kidney diseases.

Intersecting challenges of gender, race, and caregiving in healthcare

The challenges women encounter in the healthcare system, especially those burdened with caregiving responsibilities, are significantly amplified when considering the intersections of gender and racial disparities [8]. Women, particularly those from underrepresented racial and ethnic groups, often bear the disproportionate responsibility of caregiving. This additional burden can severely disrupt their ability to adhere to necessary treatment plans and maintain regular medical follow-ups, which are critical for managing chronic conditions like kidney diseases. The struggle to balance caregiving duties with personal health management is even more daunting for these women, frequently leading to delayed diagnoses and suboptimal management of their conditions due to compounded socioeconomic barriers and systemic biases [4].

Strategies for addressing gender and racial disparities in nephrology

Confronting and rectifying gender and racial disparities in nephrology necessitate a comprehensive, multi-tiered strategy. This approach must begin with efforts to enhance gender and racial diversities within nephrology research, through actively recruiting and retaining a more diverse group of participants in clinical trials and observational studies. This includes not only a higher number of women but also a more representative sample of women from various racial and ethnic backgrounds [1]. Research funders and institutions should prioritize the exploration of sex-specific and race-specific differences in kidney disease. Such a focus is crucial for developing more refined, evidence-based clinical guidelines and therapeutic interventions that are inclusive of all demographic groups.

Equipping medical professionals with the tools to recognize and address unconscious biases is pivotal in transforming healthcare culture into one that promotes inclusivity, respect, and dignity for all patients, transcending gender, race, or any other demographic distinction. Such training can lead to increased patient trust and satisfaction, which is instrumental in improving health outcomes for individuals receiving nephrology care. It is particularly crucial in nephrology, where gender and racial disparities in treatment and outcomes have been historically documented, to ensure that these biases do not continue to perpetuate inequalities in care.

Additionally, it is crucial to provide healthcare providers with extensive training designed to identify and address unconscious biases, with a particular emphasis on those related to both gender and race. These educational programs are vital for fostering an environment of awareness and sensitivity within the healthcare workforce [9]. By doing so, it ensures the delivery of equitable, patient-centric care that takes into account the unique experiences and needs of diverse patient populations. Implementing such strategies can significantly contribute to the elimination of systemic barriers and biases, promoting a more inclusive and equitable healthcare system for individuals with kidney diseases [10].

Advancing gender and racial equity in nephrology through policy and clinical practice reform

The pursuit of gender equity in nephrology calls for a thorough reevaluation and overhaul of healthcare policies and clinical guidelines to make them gender-responsive, which also involves considering racial and ethnic disparities. This includes the development and implementation of strategies that improve access to specialized care and transplantation for women, especially women of color, who are affected by kidney diseases. Additionally, it is important to integrate gender-specific and race-conscious data into clinical decision-making tools, such as treatment algorithms and risk stratification models, to ensure that these tools serve all populations equitably and effectively.

In order to rectify existing inequalities, healthcare policies must be restructured to offer more comprehensive support for women, as well as individuals from marginalized racial and ethnic groups, with kidney diseases. This could involve increasing funding for health initiatives that are specifically tailored to the needs of women and racial minorities, launching educational campaigns that are sensitive to the diverse experiences of these groups regarding treatment options and reducing financial barriers through more inclusive insurance coverage and subsidies. Such policies must take into account not only gender but also the intersectionality of race and ethnicity to effectively support all patients with kidney diseases.

Diagnostic criteria and therapeutic interventions should be refined to reflect the variances in how diseases present, respond to treatment, and the range of comorbidities that frequently diverge between men, women, and particularly women of color [11]. For instance, the management of hypertension in women with CKD needs to be sensitive to reproductive health considerations, including pregnancy, and should factor in the physiological effects of hormonal fluctuations and menopause [4,10]. This approach should also be inclusive of racial and ethnic differences that may influence the prevalence, progression, and treatment efficacy of kidney diseases.

By implementing policy changes that cater to the unique needs of women with kidney diseases, the healthcare system can make significant strides in providing personalized, effective, and equitable care [11]. This advancement is crucial for achieving the best possible health outcomes for women and establishing a standard of gender equity within healthcare. It is important to ensure that these efforts also account for racial disparities, as women of color may face additional challenges and risks due to systemic inequalities.

Moreover, there's a pressing need to refine nephrology's risk stratification frameworks by integrating factors that are uniquely significant to women. These enhancements should capture gender-specific risk factors and prognostic markers, including hormonal variations, reproductive history, and broader socioeconomic elements that disproportionately affect women, particularly those from marginalized racial and ethnic backgrounds [12]. While the CKD-EPI 2021 formula for estimating glomerular filtration rate (GFR) already incorporates gender, there is room to further tailor these models to encompass a wider array of gender-related variables. By doing so, these models will be better positioned to pinpoint women at increased risk of unfavorable outcomes. Healthcare practitioners can leverage these enhanced models to devise and apply proactive, tailored interventions more efficiently, thereby addressing the compound effects of gender and racial disparities in healthcare delivery.

Empowering women in nephrology toward gender-sensitive and inclusive healthcare practices

The shift to a gender-sensitive approach in nephrology care represents a profound overhaul of the status quo, calling for a systemic transformation of healthcare policies and clinical guidelines. This change requires a clear recognition of and a proactive response to the disparities in healthcare access, particularly those faced by women and, even more critically, by women from racial and ethnic minority groups. By integrating gender-specific insights into all aspects of clinical decision-making and acknowledging the additional challenges posed by racial disparities, healthcare systems can progress toward a practice of medicine that is both equitable and inclusive. This progression is crucial to ensure that every individual, regardless of gender or race, receives the highest standard of care for kidney diseases. A steadfast commitment to such equity is essential for enhancing the quality of nephrology services and improving health outcomes for all patients.

Patient education and empowerment are indeed pivotal in promoting an equitable healthcare landscape, particularly within nephrology [4,13]. This is especially true for women and women of color, who have historically been underrepresented in patient advocacy and decision-making roles [6]. Ensuring that women have the knowledge and the power to actively participate in their healthcare decisions is crucial. This requires providing them with access to information that respects their cultural and linguistic needs and creating an environment where their healthcare needs are heard and addressed. A personalized and comprehensive approach to care is reliant on such an empowered patient-provider relationship, one that supports patients in becoming informed and engaged advocates for their own health.

Furthermore, to truly empower women in the healthcare setting, it is critical to foster a collaborative relationship between them and their healthcare providers. Medical professionals should not only welcome but actively encourage open and active participation from women in conversations about their health. Attention must be paid to women's insights about their own preferences, values, and health objectives. This input must then be integrated into the development of personalized care plans. Such an approach is imperative to ensure that the voices of women, particularly those from marginalized communities, are not just heard but are influential in shaping their healthcare journey. By doing so, the healthcare system can move closer to a model where women’s healthcare needs are met with the respect and attention they rightly deserve.

Enhancing equitable care and supporting women across racial and ethnic divides

Providing women with the resources and support necessary to navigate the healthcare system is crucial for achieving equitable care in nephrology, and this effort must be inclusive of women from all racial and ethnic backgrounds. This includes assistance with logistical tasks such as scheduling appointments, obtaining referrals, understanding financial assistance programs, and clarifying insurance benefits. Specifically, these efforts should address the unique barriers faced by women of color, who may encounter additional systemic obstacles in accessing care. Empowering women with these tools is a critical step in enabling them to overcome the barriers to accessing care and, as a result, improving their kidney health outcomes.

The concerted effort to improve the quality of life for women with kidney diseases is fundamental in healthcare. By adopting a patient-centered approach, healthcare providers are tasked with delivering care that not only meets the highest technical standards but also encompasses compassion and responsiveness to the needs of each patient. This approach must explicitly address the needs of women from all racial and ethnic backgrounds, recognizing the intersectionality of gender and racial disparities that can affect health outcomes. In doing so, healthcare providers can contribute to better health outcomes for all patients, particularly for those who have historically faced systemic inequalities in care.

Conclusion

In conclusion, to build a more equitable healthcare landscape in nephrology, it is essential to confront and eliminate gender disparities, which also involve addressing racial and ethnic disparities. This includes bridging the research gap by ensuring the inclusion of a diverse cohort of women in clinical studies, actively working to dismantle biases within healthcare practices, and enhancing patient empowerment, especially for women of color who may face double binds of discrimination. It is a comprehensive approach that calls for the collaboration of researchers, healthcare professionals, policymakers, and patients. A unified commitment to these goals is crucial to implementing equitable healthcare practices in nephrology, assuring that every individual, regardless of their gender or racial background, receives the quality care they rightfully deserve.
